# Prospective cohort of AIDS patients screened for cryptococcal antigenaemia, pre-emptively treated and followed in Brazil

**DOI:** 10.1371/journal.pone.0219928

**Published:** 2019-07-25

**Authors:** Moara Alves Santa Bárbara Borges, João Alves de Araújo Filho, Bruno de Jesus Silva Oliveira, Isabela Silvério Moreira, Vanessa Valadares de Paula, Angélica Lima de Bastos, Renata de Bastos Ascenço Soares, Marília Dalva Turchi

**Affiliations:** 1 Institute of Tropical Pathology and Public Health of Federal University of Goiás–Goiânia, Goiás, Brazil; 2 Infectious Disease Unit, Clinical Hospital of Federal University of Goiás–Goiânia, Goiás, Brazil; 3 Pontifical Catholic University of Goiás–Goiânia, Goiás, Brazil; 4 Tropical Diseases State Hospital “Dr. Anuar Auad”–Goiânia, Goiás, Brazil; 5 State Laboratory of Public Health “Dr. Giovanni Cysneiros”–Goiânia, Goiás, Brazil; University of KwaZulu-Natal, SOUTH AFRICA

## Abstract

**Background:**

Cryptococcal meningitis has a high morbidity and mortality among AIDS population. Cryptococcal antigen (CrAg) detection is considered an independent predictor for meningitis and death. Since 2011, the World Health Organization recommends CrAg screening for people living with HIV/AIDS (PLHAs) with CD4 counts <100–200 cells/μl. Its implementation is still limited in low-middle-income countries. We aimed to estimate the prevalence and predictors of CrAg positivity in PLHAs. We also evaluated outcomes among those who were CrAg-positive.

**Methods:**

Prospective cohort conducted at an infectious diseases hospital, in Brazil. Adults with CD4 <200 cells/μl, without previous cryptococcal disease and regardless of symptoms, were enrolled from 2015 to 2018. CrAg tests were performed by LFA. Lumbar puncture was done in CrAg+ individuals and pre-emptive therapy was offered for those without meningitis.

**Results:**

Of 214 individuals recruited, 88% were antiretroviral experienced, of which only 11.6% with viral suppression. Overall, CrAg prevalence was 7.9% (95% CI, 4.7–12.4). In CD4 ≤100 cells/μl group it was 7.5% (95% CI, 4.1–12.6) and 9.1% (95% CI, 3.4–19.0) in the group with CD4 101 to 199 cells/μl (p = 0.17). Prevalence in asymptomatic subjects was 5.3% (95% CI, 1.4–13.1). One among 17 CrAg+ participants had documented meningoencephalitis and no subclinical meningitis was detected. Adherence to pre-emptive treatment was 68.7% (11/16). There were no statistically significant differences in sociodemographic, clinical or laboratory characteristics to predict CrAg positivity. No case of cryptococcal disease was diagnosed among CrAg + subjects, followed by a median of 12 months.

**Conclusions:**

CrAg screening for severely immunosuppressed PLHAs in Brazil yielded a prevalence of 7.9%. No difference was found in the prevalence of CrAg stratified by CD4 values (CD4 <100 *versus* CD4 101–199 cells/μl). No clinical nor laboratory factors predicted CrAg positivity, corroborating the need for the implementation of universal CrAg screening for PLHAs with CD4 <200 cells/μl in similar settings.

## Introduction

Cryptococcal meningoencephalitis accounts for 70% to 90% of cases of cryptococcal disease in patients with HIV; it is a life-threatening infection and the second leading cause of death in this population [1, 2]. Despite the widespread availability of antiretroviral treatment (ART), patients still present late for care, have low adherence and a significant proportion do not achieve a sustained undetectable viral load, remaining without immune recovery [3, 4]. In Brazil in 2017, around 860,000 (630,000–1,100,000) people were living with HIV, 84% of whom were aware of their status; 64% received ART and 59% had an undetectable viral load [5].

Latin America has the third highest rate of HIV/AIDS-related cryptococcal meningitis in the world, with an estimated 7,000 (3,600–11,100) people with positive antigenaemia, 5,300 (2,600–8,900) with cryptococcal meningitis and 2,400 (1,100–4,400) deaths annually [1]. A case fatality rate around 70% has been estimated in low-income countries, 40% in middle-income countries and 20%-30% in high-income countries such as the United States and Europe [1]. In Latin America, it has been reported to range from 13% to 73% [6]. The mortality rate of cryptococcosis as the primary cause of death among people living with HIV/AIDS (PLHAs) in Brazil was found to be 0.47/million inhabitants [7], reaching 30% to 62% of fatality in cryptococcal meningitis [8–10].

Since 2011, the World Health Organization (WHO) has recommended screening for cryptococcal antigen (CrAg) in people living with HIV/AIDS (PLHAs) with a CD4 count <100 cells/μl. Screening may be also considered at a higher CD4 cell count threshold of 200 cells/μl [11, 12]. The presence of CrAg in a blood sample can be identified at least 22 days to >100 days before the presentation of neurological symptoms [2, 13]. It is an independent predictor of mortality in the first 12 weeks of ART and of the development of cryptococcosis in the first year of ART [14, 15].

CrAg screening was initially recommended for regions with a prevalence of cryptococcosis ≥ 3%, allowing early identification of cryptococcal disease, even at a subclinical stage. This recommendation is reinforced by 2018 WHO Guideline, regardless of a prevalence threshold [2, 12]. The lateral flow assay (LFA) (Immuno-Mycologics Inc, Norman, OK, USA) was validated within the scope of this screening strategy, favoring its application in clinical practice [11, 16–24]. The CrAg LFA has some advantages: quick results (< 10 minutes), little training required for use and interpretation, minimal laboratory infrastructure required and no need for refrigerated storage [25]. It is cost-effective in several scenarios [19], including sites with a prevalence as low as 0.6% [26], much lower than the cut-off point suggested initially.

Since 2017, the Brazilian Guideline for Adult HIV Care [27] recommends CrAg screening in PLHAs with advanced immunosuppression and from areas with a high prevalence of cryptococcal antigenaemia. To date, CrAg screening routine implementation is still limited in Brazil and others low-middle-income countries. Therefore, this study aimed to estimate the prevalence and predictors for cryptococcal antigenaemia in PLHAs with CD4 < 200 cells/μl in the Midwest region of Brazil, and to evaluated the incidence of outcomes among those who were serum CrAg positive and were pre-emptively treated.

## Materials and methods

### Participants and study design

It is a prospective clinical cohort study conducted at the main infectious diseases public health service, responsible for caring for most PLHAs in the state of Goiás (6.7 million inhabitants), in the Midwest of Brazil. HIV assistance and ART are offered free of charge in Brazil. This study is part of a project from the Brazilian Network of Cryptococcosis. The participants were recruited and blood samples were collected from June 2015 to July 2018. They were older than 17 years, had a laboratory confirmed diagnosis of HIV and a CD4+ T lymphocyte count < 200 cells/μl, regardless of symptoms. The exclusion criterion was previous cryptococcal disease. The study protocol is available at: dx.doi.org/10.17504/protocols.io.22fggbn.

The data were obtained from clinical evaluation and from chart review. We invited hospitalized or outpatient assisted HIV-positive individuals, according to a confidential list provided by the central laboratory. Outpatients were accessed by phone calls. After giving written informed consent, the participants were interviewed, examined by a medical doctor from the research team, and had serum CrAg and other laboratory exams performed. We also reviewed the medical records and previous laboratory results of all participants in order to obtain more reliable data on time of HIV diagnosis, CD4 cell count, HIV viral load, ART history and previous opportunistic infections.

We considered the presence of fever, weight loss, asthenia, pulmonary symptoms, diarrhea or oral candidiasis as general symptoms. Neurological symptoms included headache, convulsions, changed mental state, dizziness, and visual or auditory deficit. In case suspected meningitis, lumbar puncture and CSF analysis were readily performed in association with serum antigen, according to current WHO recommendations. Serum cryptococcal antigen by LFA (Immuno-Mycologics Inc) and complementary examinations were performed, including haemogram, urea, creatinine, proteinogram, and haemoculture for fungi.

During the first month after the recruitment, all CrAg positive individuals were reevaluated at least twice by the research physician: for lumbar puncture, treatment prescription and for assessing adherence. Subsequently, the patients were followed according to routine of the health service center.

We performed a lumbar puncture on all individuals with positive serum CrAg to rule out meningeal involvement through India ink microscopy on the cerebrospinal fluid (CSF), CSF CrAg test and fungal culture. When cryptococcal meningitis was detected, the patient was treated with amphotericin B deoxycholate (0.7–1 mg/kg/day) or with a lipid formulation (3–5 mg/kg/day) associated with fluconazole (800–1,200 mg/day), because 5-fluytosine is not available in Brazil.

Pre-emptive fluconazole treatment was proposed for those with positive serum CrAg, without central nervous system involvement. The following doses were prescribed: 900 mg for 2 weeks, 450 mg for 8 to 10 weeks and a subsequent maintenance dose of 150–300 mg, according to the modified WHO recommendations [2]. At that time, only 150 mg fluconazole capsules were available in the Brazilian public health system.

In January 2019, the follow-up data of all CrAg positive participants were reassessed by chart review to measure outcomes. We investigated the rate of adherence to ART and pre-emptive therapy; the recent CD4 count and viral load; the incidence of cryptococcal disease, and the mortality rate in this group.

### Statistical analysis

The sample size was calculated using the formula: N = (Z^2^ × P[1 − P])/E^2^, where N is the sample size; Z is the level of confidence according to the standard normal distribution (for 95% level of confidence, Z = 1.96), P is the estimated prevalence of CrAg positivity among the participants (we considered P = 0.10), and E is the tolerated margin of error (proportion within 4%), resulting in a required sample of 208 participants.

Data were input into Microsoft Office Excel spreadsheets and analyzed using the Statistical Package for the Social Sciences version 23.0 (SPSS, IBM Corporation, Armonk, NY, USA). Descriptive and exploratory analyses were performed to summarize the characteristics of the individuals. Central measures (mean or median) and dispersion measures (standard deviation and interquartile rage) were calculated for continuous variables, and percentage for categorical variables. We estimated CrAg prevalence considering CD4 ≤ 100 and 101–199 cells/μl, with 95% confidence interval (CI).

Univariate analysis was performed to investigate sociodemographic, clinical, and laboratory factors potentially associated with serum CrAg positivity. Chi-squared test or the Fisher exact test were used for comparison of categorical variables and the t test for continuous variables. All tests were two-tailed, with the significance level set at <0.05

### Ethics statement

This study was approved by the institutional review board of Tropical Diseases State Hospital “Dr. Anuar Auad” (CAAE: 42429715.5.1001.0034). All participants gave written informed consent (as outlined in PLOS consent form) to publish these case details. The data collected were considered confidential and are presented in anonymous manner. The consent form used is available in an online repository.

## Results

A total of 214 individuals with CD4 cell counts < 200 cells/μl were enrolled, of whom 72.4% were male. The median age was 40 (interquartile range (IQR) 33–49) years. Most of the individuals (78.5%) were interviewed on an outpatient basis. About half of the participants were diagnosed with HIV within the previous 12 months, 40.2% had been diagnoses for more than 5 years and 68.2% had previous opportunistic disease. Before enrolment, 88.8% of the participants had started ART at some time, of whom 51.4% were regular users of this medication in the last 3 months, but only 11.6% were viral suppressed. The sociodemographic, and clinical characteristics, including the use of ART, are shown in Table 1.

**Table 1:** Sociodemographic and clinical characteristics of 214 individuals screened for serum CrAg.

Variables	Total, n (%)	CrAg positive, n (%)	CrAg negative, n (%)	p value^1^
CrAg tested	214	17 (7.9)	197 (92.1)	
Sex				
Male	155 (72.4)	14 (9.0)	141 (91.0)	0.411
Female	59 (27.6)	3 (5.1)	56 (94.9)	
Age				
Age (years), median (SD)	41.8 (10.9)	38.47 (11.4)	41.3 (10.9)	0.306
≤ 40 years	112 (52.3)	11 (9.8)	101 (90.2)	0.322
> 40 years	102 (47.7)	6 (5.9)	96 (94.1)	
Presence of symptoms				
Asymptomatic	75(35)	4 (5.3)	71 (94.7)	0.316
Any symptoms	139 (65)	13 (9.3)	126 (90.7)	
General symptoms	103 (48.1)	6 (5.8)	97 (94.2)	0.318
Neurological symptoms	87 (40.7)	10 (11.5)	77 (88.5)	0.128
Previous opportunistic diseases				
Yes	146 (68.2)	13 (8.9)	133 (91.1)	0.591
No	68 (31.8)	4 (5.9)	64 (94.1)	
HIV diagnosis				
≤12 months	101 (47.2)	7 (6.9)	94 (93.1)	0.625
>12 months	113 (52.8)	10 (8.8)	103 (91.2)	
Experience with ART^2^				
Yes	190 (88.8)	16 (8.4)	174 (91.6)	0.700
No (naive)	24 (11.2)	1 (4.2)	23 (95.8)	
Regular^3^ use of ART				
Yes	110 (51.4)	11 (10)	99 (90)	0.316
No	104 (48.6)	6 (5.8)	98 (94.2)	

^1^Level of significance. For categorical variables, the chi-squared test was used; if n<5 in any cell, the Fisher exact test was used. The t test was used for continuous variables.

^2^ART, antiretroviral therapy.

^3^regular use of ART, medication use during the last 3 months.

Overall, 73.3% had CD4 count < 100 cells/μl, 53.3% had up to 50 cells/μl, with median of 47 (IQR 20–102) cells/μl. In the CrAg-positive group, the median CD4 was 56 (IQR 27–128) cells/μl and 47 (IQR 19–102) cells/μl in the CrAg-negative group (p = 0.466). Regarding viral load, 51.4% had > 100,000 copies/ml and 25 participants had a viral load below the detection threshold (< 40 copies/ml). Characteristics related to the laboratory tests are described in Table 2. No clinical, sociodemographic or laboratory characteristics were identified as related to the positivity of cryptococcal antigenaemia.

**Table 2:** Laboratory tests of the 214 individuals screened with serum CrAg.

Tests	Total	CrAg positive, n (%)	CrAg negative, n (%)	p value
CD4				
≤ 100 cells/μl	159	12 (7.5)	147 (92.5)	0.773
> 100 cells/μl	55	5 (9.1)	50 (90.9)	
Viral load				
< 100,000 copies/ml	104	10 (9.6)	94 (90.4)	0.379
≥ 100,000 copies/ml	110	7 (6.4)	103 (93.6)	
Haemoglobin^1^				
< 12 g/dL	75	3 (4)	72 (96)	0.122
≥ 12 g/dL	136	14 (10.3)	122 (89.7)	
Hematocrit^1^				
< 35%	63	2 (3.2)	61 (96.8)	0.104
≥ 35%	148	15 (10.1)	133 (89.9)	
White blood cells^1^				
< 3,500 cells/L	56	3 (5.4)	53 (94.6)	0.568
≥ 3,500 cells/L	55	14 (9.0)	41 (141)	
Platelets^2^				
< 150×10^9^ cells/L	46	4 (8.7)	42 (91.3)	1.000
≥ 150×10^9^ cells/L	51	13 (8.1)	48 (91.9)	
Albumin^3^				
< 3.5 g/dL	45	2 (4.4)	43 (95.6)	0.372
≥ 3.5 g/dL	151	14 (9.3)	137 (90.7)	

For categorical variables, the chi-squared test was used; if n<5 in any cell, the Fisher exact test was used. The t test was used for continuous variables.

^1^Three missing values.

^2^Seven missing values.

^3^18 missing values.

Among the 214 participants, 17 presented with positive cryptococcal antigenaemia, resulting in an overall prevalence of 7.9% (95% CI, 4.8–12.2). The prevalence of CrAg was 7.5% (95% CI, 4.1–12.6) in individuals with CD4 ≤ 100 cells/μl and 9.1% (95% CI, 3.4–19.0) in those with a CD4 count between 101 and 199 cells/μl (p = 0.77).

Sixteen individuals who were CrAg positive had experience of ART but only one had an undetectable viral load. In the outpatient group (168 individuals), the prevalence of cryptococcal antigenaemia was 7.7% (95% CI, 4.4–12.6), and in the hospitalized group (46 individuals) it was 8.7% (95% CI, 2.8–19.6), without a significant difference between the groups (p = 0.76). The prevalence of CrAg among asymptomatic participants was 5.3% (95% CI, 1.4–13.1), and 9.3% (95% CI, 5.5–15.3) among symptomatic participants, with no significant difference between the groups (p = 0.316).

All individuals with positive antigenaemia underwent lumbar puncture. No subclinical cryptococcal meningitis was detected. One among 17 CrAg+ had documented symptomatic meningoencephalitis. This was demonstrated by positive India ink microscopy, positive CrAg LFA and positive culture for *Cryptococcus* spp in CSF. This patient presented severe intracranial hypertension (initial CSF pressure of 140 cmH_2_O), which improved without need for shunt, after 5 weeks of regular lumbar punctures. He received treatment with amphotericin B (first deoxycholate 50 mg/day, then lipid complex 250 mg/day, due to altered renal function) associated with fluconazole 800 mg for 6 weeks. Regular outpatient follow-up was maintained at another facility. Thirteen out 16 individuals with positive CrAg received pre-emptive induction therapy with fluconazole. Four individuals received itraconazole at a dose of 400 mg in the induction phase and maintained this antifungal therapy during consolidation and maintenance, three because of a history of histoplasmosis and one as aspergillosis treatment. Adherence to pre-emptive treatment was 68.7% (11/16).

After median follow-up of 12 months (range, 6–42 months, IQR 6–16), no further cases of cryptococcal meningitis or cryptococcosis were reported in the CrAg-positive group. The overall mortality rate was 11.7% (2/17), with no cryptococcal disease as cause of death. Even though the majority of CrAg+ was ART experienced by the time of recruitment, 94.1% were no viral suppressed (< 40 copies/ml). The median time without effective treatment was 31 (IQR 5–129) months. 10 out 17 CrAg+ individual had to change the initial ART regimen due to virological failure. Six of them had viral resistance confirmed by HIV genotyping and achieved viral suppression after switch of ART regimen. The most common antiretroviral regimens after switch were: tenofovir desoproxil and lamivudine associated with dolutegravir and / or protease inhibitors. The median time to achieve viral suppression after recruitment was 4 (IQR 2–10) months.

Most of the CrAg+ patients (88%) remained under care, and 76.4% (13/17) achieved undetectable viral load. The median CD4 count increased from 56 (IQR 27–128) cells/μl to 140 (IQR 98–140) cells/μl and 23.5% (4/17) remained with a CD4 count < 100 cells/μl. Details of the follow-up of CrAg-positive individuals are presented in Table 3. Additional details are provided in the Supporting Information (S1 File).

**Table 3:** Characteristics of CrAg-positive individuals and follow-up.

Patient	Duration of detectable viral load T1^1^ (months)	Follow-up time (months)	Cryptococcal Meningitis	T1 CD4 (cells/μl)^1^	T1 viral load (copies/ml)^1^	Regular ART on T1^1^	T2 CD4 (cells/μl)^2^	In care	T2 viral load (copies/ml)^2^	Time to viral suppression after study (months)	Regular ART on T2^2^	Treatment - definitive - pre-emptive
1	121	42	No	28	621,269	Yes	485	Yes	<40	7	Yes	Fluconazole
2	4	42	Yes	199	135	Yes	425	Yes	183	12	Yes	Amphotericin+Fluconazole
3	5	28	No	130	10,383	Yes	332	Yes	<40	15	Yes	Fluconazole
4	10	17	No	126	9,270	Yes	47	Yes	<40	10	Yes	Itraconazole
5	5	16	No	7	140,766	Yes	184	Yes	<40	2	Yes	Fluconazole
6	93	15	No	75	378,294	No	140	Yes	<40	4	Yes	Itraconazole, irregular^3^
7	257	14	No	36	4,218	No	132	Death	<40	3	Yes	Fluconazole, irregular
8	148	12	No	10	54,376	No	125	Yes	125,559	10	No	Fluconazole
9	31	12	No	37	52,2170	Yes	124	Yes	<40	6	Yes	Fluconazole
10	59	7	No	27	25,826	Yes	243	Yes	<40	2	Yes	No use
11	59	8	No	31	2,423,874	No	6	Death	1,112,547	-	No	No use
12	192	7	No	56	50	No	72	Yes	<40	4	Yes	Itraconazole
13	2	7	No	20	337,560	No	117	Yes	<40	4	Yes	Fluconazole
14	1	6	No	96	112,513	Yes	290	Yes	<40	6	Yes	Itraconazole
15	138	6	No	97	121	Yes	97	Yes	121	-	Yes	No use
16	13	6	No	158	<40	Yes	202	Yes	<40	1	Yes	No use
17	7	6	No	143	228	Yes	308	Yes	<40	1	Yes	Fluconazole

^1^At time of screening (T1 = first evaluation).

^2^At time of re-evaluation T2 = (second evaluation).

^3^Irregular use of pre-emptive treatment: <10 weeks.

## Discussion

In the present study, the overall prevalence of cryptococcal antigenaemia was 7.9% (95% CI, 4.9–12.2) and is considered high. There was no difference in the prevalence of CrAg among individuals with CD4 lower than 100 cells/μl compared with those with CD4 counts between 101 and 199 cells/μl. Most individuals were experienced with ART, however only 11.6% had an undetectable HIV viral load, reinforcing the relevance of CrAg screening among severe immunocompromised HIV individuals, regardless of ART status. The study contributed to the fact that there were no new cases of cryptococcal disease in the CrAg positive group and no death related to this disease during follow-up.

Brazil is a large country with varying morbidity profiles and scarcity of data on the prevalence of CrAg among the HIV population. The prevalence of CrAg in Brazil was 2.6% (95% CI, 1.3–4.6) in the North Region (Pará State) [28], regardless of CD4 count and symptoms. In the southeast region, it varied from 3.1% (95% CI, 1.0–7.0) in asymptomatic individuals with a CD4 count <200 cells/μl in São Paulo [29] to 11.2% (95% CI, 6.2–19.5) in Rio de Janeiro, regardless of symptoms [30]. However, comparisons between different regions should be made with caution, considering the immunosuppressive profile of the individuals recruited, the presence or absence of symptoms suggestive of cryptococcal meningitis and the use of ART. For example, in the study from Pará [28], if only participants with a CD4 count < 200 cells/μl were included, the prevalence of CrAg would be 8.3%. In the study from São Paulo., if symptomatic and asymptomatic patients were grouped, the prevalence was 11.4% (95% CI, 7.9–16.3) [29], both similar to our study.

A systematic review that aimed to assess the prevalence of cryptococcal antigenaemia found values ranging from 0.2% to 22.5%, depending on the geographic area and immune status of the HIV population studied [31]. In the mentioned systematic review, most of the studies were conducted in resource-limited settings, mainly in Africa, with only one study from Latin America. The pooled prevalence of CrAg among individuals with CD4 count <100 cells/μl was 6.4% (95% CI, 5.7–7.2), which was similar to our results. However, among people with a CD4 count between 101 and 200 cells/μl, the pooled prevalence was 2.0% (95% CI, 1.2–2.7), which was lower than the prevalence of 9.1% (95% CI, 3.4–19.0) found in our study. This may be due to the difference in endemicity of cryptococcosis in the different regions studied. Probably, in this area, there is a high cryptococcal exposure and the screening at a higher level of CD4 can be justified.

The prevalence of CrAg among those with a CD4 cell count ≤ 100 cells/μl was shown to be higher among hospitalized patients (9.8%; 95% CI, 4.0–15.5) than among patients treated at outpatient clinics (6.3%; 95% CI, 5.3–7.4), with similar confidence intervals [31]. We obtained a similar prevalence among these groups, with no statistically significant difference. The difference in results could be explained by our small sample size, with low power to detect differences (type II error), as the cited review included > 2,000 CrAg-positive cases. In addition, most of the studies screened ART-naive individuals with a recent HIV diagnosis. Our population differed in that as it comprised mostly individuals with a long-term diagnosis of HIV, without virological control and with a long period of severe immunosuppression.

Even in the subgroup of symptomatic participants, the symptoms were mostly mild and non-specific, as in a real-world scenario, and we didn’t find association with CrAg antigenaemia. We did not identify any demographic, clinical or laboratory variables that could assist in predicting CrAg positivity, reinforcing the concept that screening is needed for all individuals with advanced HIV immunosuppression regardless of clinical manifestations.

Similar to other studies [29, 30, 32, 33], our population comprised individuals with a previously established diagnosis of HIV, already ART experienced, with a history of signs and symptoms of AIDS and opportunistic diseases. Even in this scenario, the strategy of CrAg screening is still justifiable, because immunosuppression itself is a risk factor for reactivation of cryptococcal disease and the protective factor related to ART would only be relevant in the context of good adherence and viral suppression [31, 33].

No subclinical cryptococcal meningitis was detected and we did not find any new case in CrAg-positive individuals, even in those with low adherence to pre-emptive treatment. This contrasts with a recent study reporting 33% (95% CI, 21–45) of asymptomatic cryptococcal meningitis among CrAg-positive individuals and an incidence of cryptococcal meningitis during follow-up of 21.4% (95% CI, 11.6–34.4) without pre-emptive fluconazole and 5.7% (95% CI, 3.0–9.7) with pre-emptive fluconazole [34]. This is probably due to our small sample size, with few positive cases. In addition, our CrAg-positive individuals remained mostly on ART, with virological control and CD4 count >100 cell/μl in more than 75%.

Our study has some limitation, due to the use of a convenience sample and lack of the results of follow-up of CrAg negative participants. We did not use a random sampling process to select participants. In order to have a representative sample, we invited hospitalized or outpatient assisted HIV-positive individuals, according to a confidential list provided by the central laboratory. Outpatients were accessed by phone calls. However, individuals who could not be contacted or who did not come to the service, probably because of social issues or clinical status, may generate a population with different characteristics. The prevalence could be underestimated, as this population could be less adherent to ART. Due to financial restriction, titration of the CrAg LFA tests was not performed; only qualitative results were obtained. Thus, there is a risk of false-positives in tests with low titration (≤ 1:5) up to 34% [35]. This may produce a selection bias.

Our strengths are that this is the first study in Latin America that evaluates the outcomes of patients screened for CrAg and pre-emptively treated. The population differs from most studies and is composed of PLHAs already experienced in ART, non-asymptomatic and with a higher threshold for CD4 count. Our recruitment was innovative. We hope that it will be the starting point for a reflex screening strategy, since the Central Laboratory is also responsible for conducting the CD4 count tests.

## Conclusion

The prevalence of cryptococcal antigenaemia in the Midwest region of Brazil is 7.9% (95% CI, 4.9–12.2) among PLHAs with CD4<200 cells/μl, regardless of symptoms, and 5.3% (95% CI, 1.4–13.1) in asymptomatic group. There was no difference in the prevalence of CrAg among individuals with CD4 lower than 100 cells/μl compared with those with CD4 counts between 101 and 199 cells/μl. Pre-emptive treatment associated to ART adherence, viral suppression and CD4 restoration are main protective factors to avoid cryptococcal meningitis. Sociodemographic factors, duration of an HIV diagnosis, ART history, clinical or laboratory characteristics cannot predict positivity of CrAg. Even in HIV population with long time of diagnosis, experienced to ART and with poor adherence to it, the serum CrAg routine is still indicated when reassessing care, including those with a higher CD4 threshold of 200 cells/μl.

To date, the CrAg screening strategy is not routinely available in resource constrained countries. Health policies incorporating antigenic diagnostic methodologies, such as the cost-effective LFA, are necessary for the early identification of cryptococcal disease and its treatment, in similar settings.

## Supporting information

S1 File Statistical analysis. (PDF)
