# Supplementary material for: Prospective cohort of AIDS patients screened for cryptococcal antigenaemia, pre-emptively treated and followed in Brazil
Source: PLoS One. 2019 Jul 25;14(7):e0219928. doi: 10.1371/journal.pone.0219928 (PMC6658077; doi:10.1371/journal.pone.0219928)
Supplement: S1 File — (PDF) [file pone.0219928.s001.pdf]

Supplementary Information 1

Statistical analysis

Summary

Age.......... 2
Sex 4
Time of HIV diagnosis.......... 5
Recruitment Local 6
Symptoms.......... 8
Asymptomatic 8
Any symptoms.......... 8
General Symptoms.......... 8
Neurological Symptoms 10
Opportunistic Diseases.......... 13
Antiretroviral Treatment exposure and actual use.......... 16
CD4 lymphocytes count and Viral Load 17
Laboratory Exams.......... 22
Haemoglobin 22
Hematocrit 23
Platelets.......... 24
White blood cells.......... 24
Albumin 25
Blood Culture 26
CrAg-positive group.......... 26
ART exposure 26
ART before recruitment 26
ART in follow-up 27
Age.......... 27
CD4 count.......... 28
Viral Load.......... 29
Follow-up.......... 29
Regular Use of Fluconazole 29
Regular Use of Itracoconazole 30
Cryptococcosis during follow-up?.......... 30
Regular use of ART? 30
Time of HIV diagnosis, ART and CD4 during follow-up 30
Outcomes for CrAg-positive patients after the recruitment 32
Characteristics of CrAg-positive individuals and follow-up.......... 33
Characteristics of ART and virological control in CrAg-positive individuals.......... 34

Supplementary Information 1

Statistical analysis

Age

		Frequency	Percent
Valid	≤40 years	112	52,3
	> 40 years	102	47,7
	Total	214	100,0

N	Valid	214
	Missing	0
Mean		41,13
Median		40,00
Minimum		18
Maximum		78
Percentiles	25	33,00
	50	40,00
	75	49,00

		CRAG RESULT		Total
		Reagent	Unreactive	
Age categoriezed by 40 Y	≤40 years	11	101	112
	> 40 years	6	96	102
Total		17	197	214

Age

CRAG RESULT	Mean	N	Std. Deviation
Reagent	38,47	17	11,402
Unreactive	41,36	197	11,102
Total	41,13	214	11,126

Supplementary Information 1

Statistical analysis

Differences in age averages between groups reagent and nonreactive

Levene's Test for Equality of Variances						
F	Sig.	t	df	Sig. (2-tailed)	95% Confidence Interval of the Difference	
					Lower	Upper
,011	,916	-1,026	212	,306	-8,428	2,659
		-1,003	18,715	,329	-8,911	3,142

Age X CrAg

			CRAG RESULT		Total
			Reagent	Unreactive	
Age categorized	≤40years	Count	11	101	112
		% within Age categorized	9,8%	90,2%	100,0%
		% within CRAG RESULT	64,7%	51,3%	52,3%
	> 40years	Count	6	96	102
		% within Age categorized	5,9%	94,1%	100,0%
		% within CRAG RESULT	35,3%	48,7%	47,7%
Total		Count	17	197	214
		% within Age categorized	7,9%	92,1%	100,0%
		% within CRAG RESULT	100,0%	100,0%	100,0%

Chi-Square Tests for Age categorized by 40 years

	Value	df	Asymp. Sig. (2-sided)	Exact Sig. (2-sided)	Exact Sig. (1-sided)
Pearson Chi-Square	1,133[a]	1	,287		
Continuity Correction[b]	,658	1	,417		
Likelihood Ratio	1,152	1	,283		
Fisher's Exact Test				,322	,209
Linear-by-Linear Association	1,127	1	,288		
N of Valid Cases	214				

a. 0 cells (0,0%) have expected count less than 5. The minimum expected count is 8,10.

b. Computed only for a 2x2 table

Supplementary Information 1

Statistical analysis

Sex

		Frequency	Percent
Valid	Male	155	72,4
	Female	59	27,6
	Total	214	100,0

Sex * CRAG RESULT Crosstabulation

			CRAG RESULT		Total
			Reagent	Unreactive	
Sex	Male	Count	14	141	155
		% within Sex	9,0%	91,0%	100,0%
		% within CRAG RESULT	82,4%	71,6%	72,4%
	Female	Count	3	56	59
		% within Sex	5,1%	94,9%	100,0%
		% within CRAG RESULT	17,6%	28,4%	27,6%
Total		Count	17	197	214
		% within Sex	7,9%	92,1%	100,0%
		% within CRAG RESULT	100,0%	100,0%	100,0%

Chi-Square Tests

	Value	df	Asymp. Sig. (2-sided)	Exact Sig. (2-sided)	Exact Sig. (1-sided)
Pearson Chi-Square	,911[a]	1	,340		
Continuity Correction[b]	,451	1	,502		
Likelihood Ratio	,990	1	,320		
Fisher's Exact Test				,411	,258
Linear-by-Linear Association	,906	1	,341		
N of Valid Cases	214				

a. 1 cells (25,0%) have expected count less than 5. The minimum expected count is 4,69.

b. Computed only for a 2x2 table

Supplementary Information 1

Statistical analysis

Time of HIV diagnosis

Previous HIV Diagnosis

		Frequency	Percent
Valid	Yes	209	97,7
	No	5	2,3
	Total	214	100,0

		Frequency	Percent
Valid	< 1 year	101	47,2
	1-5 years	27	12,6
	> 5 years	86	40,2
	Total	214	100,0

Time of HIV diagnosis categorized * CRAG RESULT

Crosstabulation

Count

		CRAG RESULT		Total
		Reagent	Unreactive	
Time of HIV diagnosis categorized	< 1 year	7	94	101
	1-5 years	4	23	27
	> 5 years	6	80	86
Total		17	197	214

Time of HIV diagnosis categorized by 12 months

		Frequency	Percent	Valid Percent	Cumulative Percent
Valid	≤12 months	101	47,2	47,2	47,2
	> 12 months	113	52,8	52,8	100,0
	Total	214	100,0	100,0	

Time of HIV CAtegorized X CRAG RESULT Crosstabulation

Count

		CRAG RESULT		Total
		Reagent	Unreactive	
TempoHIVCAteg2	≤12 months	7	94	101
	> 12 months	10	103	113
Total		17	197	214

Supplementary Information 1

Statistical analysis

Chi-Square Tests: Time of HIV categorized by 12 months X CRAG RESULT

	Value	df	Asymp. Sig. (2-sided)	Exact Sig. (2-sided)	Exact Sig. (1-sided)
Pearson Chi-Square	,269[a]	1	,604		
Continuity Correction[b]	,070	1	,791		
Likelihood Ratio	,270	1	,603		
Fisher's Exact Test				,625	,397
Linear-by-Linear Association	,267	1	,605		
N of Valid Cases	214				

a. 0 cells (0,0%) have expected count less than 5. The minimum expected count is 8,02.

b. Computed only for a 2x2 table

Recruitment Local

		Frequency	Percent
Valid	Ambulatory	168	78,5
	Nursery	27	12,6
	Emergency Dep	14	6,5
	Other	5	2,3
	Total	214	100,0

		Frequency	Percent
Valid	Outpatient	168	78,5
	Inpatient	46	21,5
	Total	214	100,0

Supplementary Information 1

Statistical analysis

			CRAG RESULT		Total
			Reactive	Non-Reactive	
localestudo2	Outpatient	Count	13	155	168
		% within local	7,7%	92,3%	100,0%
		% within CRAG	76,5%	78,7%	78,5%
	Inpatient	Count	4	42	46
		% within local	8,7%	91,3%	100,0%
		% within CRAG	23,5%	21,3%	21,5%
Total		Count	17	197	214
		% within local	7,9%	92,1%	100,0%
		% within CRAG	100,0%	100,0%	100,0%

Chi-Square Tests

	Value	df	Asymp. Sig. (2-sided)	Exact Sig. (2-sided)	Exact Sig. (1-sided)
Pearson Chi-Square	,045[a]	1	,831		
Continuity Correction[b]	,000	1	1,000		
Likelihood Ratio	,044	1	,833		
Fisher's Exact Test				,765	,517
Linear-by-Linear Association	,045	1	,832		
N of Valid Cases	214				

a. 1 cells (25,0%) have expected count less than 5. The minimum expected count is 3,65.

b. Computed only for a 2x2 table

Supplementary Information 1
Statistical analysis

Symptoms

Symptomatic (general or neurological)

		CRAG RESULT		Total
		Reagent	Unreactive	
	Symptomatic	13	126	139
	Asymptomatic	4	71	75
Total		17	197	214

Asymptomatic

		Frequency	Percent
Valid	Reagent	4	5,3
	Unreactive	71	94,7
	Total	75	100,0

Any symptoms

General Symptoms

		Frequency	Percent
Valid	Yes	103	48,1
	No	111	51,9
	Total	214	100,0

Neurological Symptoms

		Frequency	Percent
Valid	Yes	87	40,7
	No	127	59,3
	Total	214	100,0

General Symptoms

Crosstab General Symptoms X CRAG RESULT

		CRAG RESULT		Total
		Reagent	Unreactive	
General symptoms	Yes	6	97	103
	No	11	100	111
Total		17	197	214

Supplementary Information 1

Statistical analysis

Chi-Square Tests: General Symptoms X CRAG RESULT

	Value	df	Asymp. Sig. (2-sided)	Exact Sig. (2-sided)	Exact Sig. (1-sided)
Pearson Chi-Square	1,219[a]	1	,270		
Continuity Correction[b]	,724	1	,395		
Likelihood Ratio	1,239	1	,266		
Fisher's Exact Test				,318	,198
Linear-by-Linear Association	1,213	1	,271		
N of Valid Cases	214				

a. 0 cells (0,0%) have expected count less than 5. The minimum expected count is 8,18.

b. Computed only for a 2x2 table

FEVER

		Frequency	Percent	Valid Percent	Cumulative Percent
Valid	Yes	41	19,2	19,2	19,2
	No	173	80,8	80,8	100,0
	Total	214	100,0	100,0	

WEIGTH LOSS

		Frequency	Percent	Valid Percent	Cumulative Percent
Valid	Yes	43	20,1	20,1	20,1
	No	171	79,9	79,9	100,0
	Total	214	100,0	100,0	

DIARRHEA

		Frequency	Percent	Valid Percent	Cumulative Percent
Valid	Yes	15	7,0	7,0	7,0
	No	199	93,0	93,0	100,0
	Total	214	100,0	100,0	

PULMONARY SYMPTOMS

		Frequency	Percent	Valid Percent	Cumulative Percent
Valid	Yes	44	20,6	20,6	20,6

	No	170	79,4	79,4	100,0
	Total	214	100,0	100,0	

SKIN INJURIES

		Frequency	Percent	Valid Percent	Cumulative Percent
Valid	Yes	21	9,8	9,8	9,8
	No	193	90,2	90,2	100,0
	Total	214	100,0	100,0	

ASTENIA

		Frequency	Percent	Valid Percent	Cumulative Percent
Valid	Yes	34	15,9	15,9	15,9
	No	180	84,1	84,1	100,0
	Total	214	100,0	100,0	

ORAL MONILLYASE

		Frequency	Percent	Valid Percent	Cumulative Percent
Valid	Yes	11	5,1	5,1	5,1
	No	203	94,9	94,9	100,0
	Total	214	100,0	100,0	

Neurological Symptoms

Neurological Symptoms X CRAG RESULT

Count

		CRAG RESULT		Total
		Reagent	Unreactive	
Neurological symptoms	Yes	10	77	87
	No	7	120	127
Total		17	197	214

Supplementary Information 1

Statistical analysis

Chi-Square Tests: NeurologicalSymptoms X CRAG RESULT

	Value	df	Asymp. Sig. (2-sided)	Exact Sig. (2-sided)	Exact Sig. (1-sided)
Pearson Chi-Square	2,527[a]	1	,112		
Continuity Correction[b]	1,775	1	,183		
Likelihood Ratio	2,473	1	,116		
Fisher's Exact Test				,128	,092
Linear-by-Linear Association	2,515	1	,113		
N of Valid Cases	214				

a. 0 cells (0,0%) have expected count less than 5. The minimum expected count is 6,91.

b. Computed only for a 2x2 table

HEADACHE

		Frequency	Percent	Valid Percent	Cumulative Percent
Valid	Yes	51	23,8	23,8	23,8
	No	163	76,2	76,2	100,0
	Total	214	100,0	100,0	

SEIZURE

		Frequency	Percent	Valid Percent	Cumulative Percent
Valid	Yes	7	3,3	3,3	3,3
	No	207	96,7	96,7	100,0
	Total	214	100,0	100,0	

SOMNOLENCE

		Frequency	Percent	Valid Percent	Cumulative Percent
Valid	Yes	6	2,8	2,8	2,8
	No	208	97,2	97,2	100,0
	Total	214	100,0	100,0	

MENTAL CONFUSION

	Frequency	Percent	Valid Percent	Cumulative Percent

Valid	Yes	7	3,3	3,3	3,3
	No	207	96,7	96,7	100,0
	Total	214	100,0	100,0	

PARAPARESIA

		Frequency	Percent	Valid Percent	Cumulative Percent
Valid	Yes	3	1,4	1,4	1,4
	No	211	98,6	98,6	100,0
	Total	214	100,0	100,0	

ESFINCTERIAN CHANGE

		Frequency	Percent	Valid Percent	Cumulative Percent
Valid	Yes	1	,5	,5	,5
	No	213	99,5	99,5	100,0
	Total	214	100,0	100,0	

VISUAL CHANGE

		Frequency	Percent	Valid Percent	Cumulative Percent
Valid	Yes	8	3,7	3,7	3,7
	No	206	96,3	96,3	100,0
	Total	214	100,0	100,0	

HEARING CHANGE

		Frequency	Percent	Valid Percent	Cumulative Percent
Valid	Yes	5	2,3	2,3	2,3
	No	209	97,7	97,7	100,0
	Total	214	100,0	100,0	

MEMORY CHANGE

		Frequency	Percent	Valid Percent	Cumulative Percent
Valid	Yes	24	11,2	11,2	11,2

	No	190	88,8	88,8	100,0
	Total	214	100,0	100,0	

DISARTRIA

		Frequency	Percent	Valid Percent	Cumulative Percent
Valid	Yes	4	1,9	1,9	1,9
	No	210	98,1	98,1	100,0
	Total	214	100,0	100,0	

DIZZINESS/VERTIGO

		Frequency	Percent	Valid Percent	Cumulative Percent
Valid	Yes	15	7,0	7,0	7,0
	No	199	93,0	93,0	100,0
	Total	214	100,0	100,0	

VOMITING

		Frequency	Percent	Valid Percent	Cumulative Percent
Valid	Yes	4	1,9	1,9	1,9
	No	210	98,1	98,1	100,0
	Total	214	100,0	100,0	

Opportunistic Diseases

Previous opportunistic diseases

		Frequency	Percent
Valid	Yes	146	68,2
	No	68	31,8
	Total	214	100,0

ORAL CANDIDIASIS

		Frequency	Percent	Valid Percent	Cumulative Percent

Supplementary Information 1

Statistical analysis

Valid	Yes	42	19,6	19,6	19,6
	No	172	80,4	80,4	100,0
	Total	214	100,0	100,0	

PNEUMOCISTOSIS

		Frequency	Percent	Valid Percent	Cumulative Percent
Valid	Yes	34	15,9	15,9	15,9
	No	180	84,1	84,1	100,0
	Total	214	100,0	100,0	

HISTOPLASMOSIS

		Frequency	Percent	Valid Percent	Cumulative Percent
Valid	Yes	27	12,6	12,6	12,6
	No	187	87,4	87,4	100,0
	Total	214	100,0	100,0	

PULMONARY TUBERCULOSIS

		Frequency	Percent	Valid Percent	Cumulative Percent
Valid	Yes	17	7,9	7,9	7,9
	No	197	92,1	92,1	100,0
	Total	214	100,0	100,0	

HERPES ZOSTER

		Frequency	Percent	Valid Percent	Cumulative Percent
Valid	Yes	5	2,3	2,3	2,3
	No	209	97,7	97,7	100,0
	Total	214	100,0	100,0	

CNS TOXOPLASMOSIS

Supplementary Information 1

Statistical analysis

		Frequency	Percent	Valid Percent	Cumulative Percent
Valid	Yes	41	19,2	19,2	19,2
	No	173	80,8	80,8	100,0
	Total	214	100,0	100,0	

CMV

		Frequency	Percent	Valid Percent	Cumulative Percent
Valid	Yes	22	10,3	10,3	10,3
	No	192	89,7	89,7	100,0
	Total	214	100,0	100,0	

KAPOSI SARCOMA

		Frequency	Percent	Valid Percent	Cumulative Percent
Valid	Yes	6	2,8	2,8	2,8
	No	208	97,2	97,2	100,0
	Total	214	100,0	100,0	

PREVIOUS OPPORTUNISTIC DISEASES X CRAG RESULT

Crosstabulation

Count

		CRAG RESULT		Total
		Reagent	Unreactive	
TEVE DOENÇA OPORTUNISTA ASSOCIADA AO HIV	Yes	13	133	146
	No	4	64	68
Total		17	197	214

Chi-Square Tests

	Value	df	Asymp. Sig. (2-sided)	Exact Sig. (2-sided)	Exact Sig. (1-sided)
Pearson Chi-Square	,579[a]	1	,447		
Continuity Correction[b]	,240	1	,624		
Likelihood Ratio	,609	1	,435		
Fisher's Exact Test				,591	,321

Linear-by-Linear Association	,577	1	,448		
N of Valid Cases	214				

a. 0 cells (0,0%) have expected count less than 5. The minimum expected count is 5,40.

b. Computed only for a 2x2 table

Antiretroviral Treatment exposure and actual use

Experience with ART

		Frequency	Percent
Valid	Yes	190	88,8
	Naive	24	11,2
	Total	214	100,0

ART actual regular use

		Frequency	Percent
Valid	Yes	110	51,4
	No	104	48,6
	Total	214	100,0

regular use of ART, medication use during the last 3 months.

ART Experience

			CRAG RESULT		Total
			Reagent	Unreactive	
EXPERIENCE WITH ART	Yes	Count	16	174	190
		% within ART EXPERIENCE	8,4%	91,6%	100,0%
		% within CRAG RESULT	94,1%	88,3%	88,8%
	Naive	Count	1	23	24
		% within ART EXPERIENCE	4,2%	95,8%	100,0%
		% within CRAG RESULT	5,9%	11,7%	11,2%
Total		Count	17	197	214
		% within ART EXPERIENCE	7,9%	92,1%	100,0%
		% within CRAG RESULT	100,0%	100,0%	100,0%

Chi-Square Tests: ART EXPERIENCE X CRAG RESULT

	Value	df	Asymp. Sig. (2-sided)	Exact Sig. (2-sided)	Exact Sig. (1-sided)
Pearson Chi-Square	,527[a]	1	,468		

Continuity Correction[b]	,106	1	,745		
Likelihood Ratio	,617	1	,432		
Fisher's Exact Test				,700	,407
Linear-by-Linear Association	,525	1	,469		
N of Valid Cases	214				

a. 1 cells (25,0%) have expected count less than 5. The minimum expected count is 1,91.

b. Computed only for a 2x2 table

Crosstab: REGULAR ART X CRAG RESULT

			CRAG RESULT		Total
			Reagent	Unreactive	
REGULAR USE OF ART DURING RECUITMENT	Yes	Count	11	99	110
		% within REGULAR ART	10,0%	90,0%	100,0%
		% within CRAG RESULT	64,7%	50,3%	51,4%
	No	Count	6	98	104
		% within REGULAR ART	5,8%	94,2%	100,0%
		% within CRAG RESULT	35,3%	49,7%	48,6%
Total		Count	17	197	214
		% within REGULAR ART	7,9%	92,1%	100,0%
		% within CRAG RESULT	100,0%	100,0%	100,0%

Chi-Square Tests

	Value	df	Asymp. Sig. (2-sided)	Exact Sig. (2-sided)	Exact Sig. (1-sided)
Pearson Chi-Square	1,308[a]	1	,253		
Continuity Correction[b]	,794	1	,373		
Likelihood Ratio	1,329	1	,249		
Fisher's Exact Test				,316	,187
Linear-by-Linear Association	1,302	1	,254		
N of Valid Cases	214				

a. 0 cells (0,0%) have expected count less than 5. The minimum expected count is 8,26.

b. Computed only for a 2x2 table

CD4 lymphocytes count and Viral Load

Supplementary Information 1

Statistical analysis

RECRUITMENT CD4 COUNT

N	Valid	214
	Missing	0
Mean		65,74
Median		47,00
Std. Deviation		54,813
Minimum		1
Maximum		199
Percentiles	25	19,75
	50	47,00
	75	102,00

Statistics

CD4 count - CRAG Positive

N	Valid	17
	Missing	0
Mean		75,06
Median		56,00
Std. Deviation		58,678
Minimum		7
Maximum		199
Percentiles	25	27,50
	50	56,00
	75	128,00

CD4 count - CRAG

N	Valid	197
	Missing	0
Mean		64,93
Median		47,00
Std. Deviation		54,551
Minimum		1
Maximum		197
Percentiles	25	19,00
	50	47,00
	75	102,00

Supplementary Information 1

Statistical analysis

RECRUITMENT VIRAL LOAD

N	Valid	214
	Missing	0
Mean		388645,08
Median		104188,00
Minimum		0
Maximum		10000000
Percentiles	25	2330,50
	50	104188,00
	75	376445,25

Supplementary Information 1

Statistical analysis

CD4 CATEGORIZED BY 100

		Frequency	Percent
Valid	≤100	159	74,3
	>100	55	25,7
	Total	214	100,0

CD4 COUNT STATISTICS

	CRAG RESULT	N	Mean	Std. Deviation	Std. Error Mean
CD4 COUNT	Reagent	17	75,06	58,678	14,231
	Unreactive	197	64,93	54,551	3,887

Crosstabulation: CD4 CATEGORIZED BY 100 X CRAG RESULT

			CRAG RESULT		Total
			Reagent	Unreactive	
CD4 COUNT	≤100	Count	12	147	159
		% within CD4 100	7,5%	92,5%	100,0%
		% within CRAG RESULT	70,6%	74,6%	74,3%
	>100	Count	5	50	55
		% within CD4 100	9,1%	90,9%	100,0%
		% within CRAG RESULT	29,4%	25,4%	25,7%
Total		Count	17	197	214
		% within CD4 100	7,9%	92,1%	100,0%
		% within CRAG RESULT	100,0%	100,0%	100,0%

Chi-Square Tests: CD4 CATEGORIZED BY 100 X CRAG RESULT

	Value	df	Asymp. Sig. (2-sided)	Exact Sig. (2-sided)	Exact Sig. (1-sided)
Pearson Chi-Square	,133[a]	1	,715		
Continuity Correction[b]	,006	1	,940		
Likelihood Ratio	,130	1	,719		
Fisher's Exact Test				,773	,454
Linear-by-Linear Association	,133	1	,716		
N of Valid Cases	214				

a. 1 cells (25,0%) have expected count less than 5. The minimum expected count is 4,37.

b. Computed only for a 2x2 table

Supplementary Information 1

Statistical analysis

VIRAL LOAD CATEGORIZED

		Frequency	Percent
Valid	≤100,000	104	48,6
	>100,000	110	51,4
	Total	214	100,0

VIRAL LOAD STATISTIC

N	Valid	214
	Missing	0
Mean		388645,08
Median		104188,00
Minimum		0
Maximum		10000000
Percentiles	25	2330,50
	50	104188,00
	75	376445,25

Crosstabulation: VIRAL LOAD CATEGORIZED X CRAG RESULT

			CRAG RESULT		Total
			Reagent	Unreactive	
VL	≤100thous	Count	10	94	104
		% within VL	9,6%	90,4%	100,0%
		% within CrAg	58,8%	47,7%	48,6%
		% of Total	4,7%	43,9%	48,6%
	>100thous	Count	7	103	110
		% within VL	6,4%	93,6%	100,0%
		% within CrAg	41,2%	52,3%	51,4%
		% of Total	3,3%	48,1%	51,4%
Total		Count	17	197	214
		% within CVCAteg	7,9%	92,1%	100,0%
		% within CrAG	100,0%	100,0%	100,0%
		% of Total	7,9%	92,1%	100,0%

Supplementary Information 1

Statistical analysis

Chi-Square Tests

	Value	df	Asymp. Sig. (2-sided)	Exact Sig. (2-sided)	Exact Sig. (1-sided)
Pearson Chi-Square	,773[a]	1	,379		
Continuity Correction[b]	,392	1	,531		
Likelihood Ratio	,775	1	,379		
Fisher's Exact Test				,453	,266
Linear-by-Linear Association	,769	1	,380		
N of Valid Cases	214				

a. 0 cells (0,0%) have expected count less than 5. The minimum expected count is 8,26.

b. Computed only for a 2x2 table

Laboratory Exams

		Haemoglobin	Hematocrit	WBC	Platelets	Urea	Creatinin	Albumin
N	Valid	211	211	211	207	210	211	196
	Missing	3	3	3	7	4	3	18
Mean		12,667	38.1	5,164	206,070	28	0.99	3.8
Median		12,800	38.9	4,860	198,000	26	0.94	4.0
Minimum		5,7	16,2	500	13800	8	0.35	0.8
Maximum		18,0	54,8	21500	486000	89	3,30	5.1
Percentiles	25	11,300	34,000	3410,00	154000,00	21,00	0.80	3.5
	50	12,800	38,900	4860,00	198000,00	26,00	0.94	4.0
	75	14,500	43,300	6400,00	244000,00	33,00	1.13	4.4

Haemoglobin

Crosstab: Haemoglobin categorized by 12 x CRAG RESULT

			CRAG RESULT		Total
			Reagent	Unreactive	
HbCAteg	<12 g/dL	Count	3	72	75
		% within HbCAteg	4,0%	96,0%	100,0%
		% within CRAG RESULT	17,6%	37,1%	35,5%
	≤12 g/dL	Count	14	122	136
		% within HbCAteg	10,3%	89,7%	100,0%
		% within CRAG RESULT	82,4%	62,9%	64,5%
Total		Count	17	194	211
		% within HbCAteg	8,1%	91,9%	100,0%
		% within CRAG RESULT	100,0%	100,0%	100,0%

Supplementary Information 1

Statistical analysis

Chi-Square Tests: Haemoglobin categorized by 12 x CRAG RESULT

	Value	df	Asymp. Sig. (2-sided)	Exact Sig. (2-sided)	Exact Sig. (1-sided)
Pearson Chi-Square	2,585[a]	1	,108		
Continuity Correction[b]	1,805	1	,179		
Likelihood Ratio	2,867	1	,090		
Fisher's Exact Test				,122	,086
Linear-by-Linear Association	2,573	1	,109		
N of Valid Cases	211				

a. 0 cells (0,0%) have expected count less than 5. The minimum expected count is 6,04.

b. Computed only for a 2x2 table

Hematocrit

Crosstab: Hematocrit categorized by 35 x CRAG RESULT

			CRAG RESULT		Total
			Reagent	Unreactive	
HtCAteg	<35%	Count	2	61	63
		% within HtCAteg	3,2%	96,8%	100,0%
		% within CRAG RESULT	11,8%	31,4%	29,9%
	≤35%	Count	15	133	148
		% within HtCAteg	10,1%	89,9%	100,0%
		% within CRAG RESULT	88,2%	68,6%	70,1%
Total		Count	17	194	211
		% within HtCAteg	8,1%	91,9%	100,0%
		% within CRAG RESULT	100,0%	100,0%	100,0%

Chi-Square Tests: Hematocrit categorized by 35 x CRAG RESULT

	Value	df	Asymp. Sig. (2-sided)	Exact Sig. (2-sided)	Exact Sig. (1-sided)
Pearson Chi-Square	2,890[a]	1	,089		
Continuity Correction[b]	2,027	1	,155		
Likelihood Ratio	3,390	1	,066		
Fisher's Exact Test				,104	,071
Linear-by-Linear Association	2,876	1	,090		
N of Valid Cases	211				

a. 0 cells (0,0%) have expected count less than 5. The minimum expected count is 5,08.

b. Computed only for a 2x2 table

Supplementary Information 1

Statistical analysis

Platelets

Crosstab: Platelets categorized by 150 X CRAG RESULT

			CRAG RESULT		Total
			Reagent	Unreactive	
PlaqCateg	<150 x 10^3	Count	4	42	46
		% within PlaqCateg	8,7%	91,3%	100,0%
		% within CRAG RESULT	23,5%	22,1%	22,2%
	≥150 x 10^3	Count	13	148	161
		% within PlaqCateg	8,1%	91,9%	100,0%
		% within CRAG RESULT	76,5%	77,9%	77,8%
Total		Count	17	190	207
		% within PlaqCateg	8,2%	91,8%	100,0%
		% within CRAG RESULT	100,0%	100,0%	100,0%

Chi-Square Tests: Platelets categorized by 150 X CRAG RESULT

	Value	df	Asymp. Sig. (2-sided)	Exact Sig. (2-sided)	Exact Sig. (1-sided)
Pearson Chi-Square	,018[a]	1	,892		
Continuity Correction[b]	,000	1	1,000		
Likelihood Ratio	,018	1	,893		
Fisher's Exact Test				1,000	,548
Linear-by-Linear Association	,018	1	,893		
N of Valid Cases	207				

a. 1 cells (25,0%) have expected count less than 5. The minimum expected count is 3,78.

b. Computed only for a 2x2 table

White blood cells

Crosstab: WBC Categorized by 3,500 X CRAG RESULT

			CRAG RESULT		Total
			Reagent	Unreactive	
WBC	<3,500 cells/L	Count	3	53	56
		% within WBC	5,4%	94,6%	100,0%
		% within CRAG RESULT	17,6%	27,3%	26,5%
	≤3,500 cells/L	Count	14	141	155
		% within WBC	9,0%	91,0%	100,0%
		% within CRAG RESULT	82,4%	72,7%	73,5%
Total		Count	17	194	211
		% within WBC	8,1%	91,9%	100,0%
		% within CRAG RESULT	100,0%	100,0%	100,0%

Supplementary Information 1

Statistical analysis

Chi-Square Tests: WBC Categorized by 3,500 X CRAG RESULT

	Value	df	Asymp. Sig. (2-sided)	Exact Sig. (2-sided)	Exact Sig. (1-sided)
Pearson Chi-Square	,750[a]	1	,386		
Continuity Correction[b]	,336	1	,562		
Likelihood Ratio	,811	1	,368		
Fisher's Exact Test				,568	,291
Linear-by-Linear Association	,747	1	,388		
N of Valid Cases	211				

a. 1 cells (25,0%) have expected count less than 5. The minimum expected count is 4,51.

b. Computed only for a 2x2 table

Albumin

Crosstab: Albumin categorized by 3.5 X CRAG RESULT

			CRAG RESULT		Total
			Reagent	Unreactive	
AlbCateg	<3.5 g/dL	Count	2	43	45
		% within AlbCateg	4,4%	95,6%	100,0%
		% within CRAG RESULT	12,5%	23,9%	23,0%
	≤3.5 g/dL	Count	14	137	151
		% within AlbCateg	9,3%	90,7%	100,0%
		% within CRAG RESULT	87,5%	76,1%	77,0%
Total		Count	16	180	196
		% within AlbCateg	8,2%	91,8%	100,0%
		% within CRAG RESULT	100,0%	100,0%	100,0%

Chi-Square Tests: Albumin categorized by 3.5 X CRAG RESULT

	Value	df	Asymp. Sig. (2-sided)	Exact Sig. (2-sided)	Exact Sig. (1-sided)
Pearson Chi-Square	1,078[a]	1	,299		
Continuity Correction[b]	,530	1	,467		
Likelihood Ratio	1,220	1	,269		
Fisher's Exact Test				,372	,242
Linear-by-Linear Association	1,072	1	,300		
N of Valid Cases	196				

a. 1 cells (25,0%) have expected count less than 5. The minimum expected count is 3,67.

b. Computed only for a 2x2 table

Supplementary Information 1

Statistical analysis

Blood Culture

Blood culture

		Frequency	Percent	Valid Percent	Cumulative Percent
Valid	Negative	203	94,9	94,9	94,9
	Positive Cryptococcus	2	,9	,9	95,8
	Positive Histoplasma	6	2,8	2,8	98,6
	Positive Candida	2	,9	,9	99,5
	Not colected	1	,5	,5	100,0
	Total	214	100,0	100,0	

CrAg-positive group

ART exposure

Previous HIV diagnosis?

		Frequency	Percent	Valid Percent	Cumulative Percent
Valid	Yes	17	100,0	100,0	100,0

ART before recruitment

		Frequência	Porcentagem
Válido	TDF + 3TC + ATV + RTV	3	17,6
	TDF+ 3TC + EFV	5	29,4
	TDF + 3TC + DTG	4	23,5
	TDF + 3TC + IP + RTV + DTG	4	23,5
	NO ART	1	5,9
	Total	17	100,0

Supplementary Information 1

Statistical analysis

ART in follow-up

		Frequência	Porcentagem
Válido	TDF + 3TC + ATV + RTV	3	17,6
	TDF + 3TC + ATV + RTV + DTG	1	5,9
	TDF + 3TC + DRV + RTV	2	11,8
	TDF + 3TC + DRV/R + DTG	5	29,4
	TDF + 3TC + DTG	5	29,4
	TDF + 3TC + EFZ	1	5,9
	Total	17	100,0

Age

Statistics

Age

N	Valid	17
	Missing	0
Mean		38,471
Median		39,000
Minimum		18,0
Maximum		57,0
Percentiles	25	29,000
	50	39,000
	75	49,500

Age categorized by 40 years

		Frequency	Percent
Valid	≤40	11	64,7
	>40	6	35,3
	Total	17	100,0

Supplementary Information 1

Statistical analysis

CD4 count

CD4 count at recruitment

N	Valid	17
	Missing	0
Mean		75,059
Median		56,000
Minimum		7,0
Maximum		199,0
Percentiles	25	27,500
	50	56,000
	75	128,000

CD4 categorized by 100

		Frequency	Percent
Valid	≤100	12	70,6
	>100	5	29,4
	Total	17	100,0

CD4 count after follow up

N	Valid	17
	Missing	0
Mean		194,588
Median		140,000
Minimum		6,0
Maximum		485,0
Percentiles	25	98,000
	50	140,000
	75	299,000

Supplementary Information 1

Statistical analysis

Viral Load

Statistics

Viral Load

N	Valid	17
	Missing	0
Mean		273003,118
Median		25826,000
Minimum		,0
Maximum		2423874,0
Percentiles	25	181,500
	50	25826,000
	75	357927,000

VL categorized by 100

		Frequency	Percent
Valid	≤100thous	10	58,8
	>100thous	7	41,2
	Total	17	100,0

Follow-up

		Frequency	Percent
Valid	Yes	15	88,2
	Óbito	2	11,8
	Total	17	100,0

Regular Use of Fluconazole

		Frequency	Percent	Valid Percent
Valid	Yes	8	47,1	61,5
	No	5	29,4	38,5
	Total	13	76,5	100,0
Missing	System	4	23,5	
Total		17	100,0	

Supplementary Information 1

Statistical analysis

Regular Use of Itracoconazole

		Frequency	Percent	Valid Percent
Valid	Yes	3	17,6	60,0
	No	2	11,8	40,0
	Total	5	29,4	100,0
Missing	System	12	70,6	
Total		17	100,0	

Cryptococcosis during follow-up?

		Frequency	Percent	Valid Percent
Valid	Yes	1	5,9	5,9
	No	16	94,1	94,1
	Total	17	100,0	100,0

Regular use of ART?

		Frequency	Percent	Valid Percent
Valid	Yes	14	82,4	82,4
	No	1	5,9	5,9
	óbito	2	11,8	11,8
	Total	17	100,0	100,0

Time of HIV diagnosis, ART and CD4 during follow-up

	Media	Median	Minimum	Maximum	Percent25	Percent75	Percent100
Time of HIV diagnosis (months)	67.6 (±78)	31	1	257	5	31	129.5
Time of HIV diagnosis (years)	5.1 (±6.5)	2	0	21	0	2	10.5
Time to first ARV (months)	33 (±53)	3	0	175	0	3	65
Time to undetectable VL (Years) - life	5.3 (±6.7)	1	0	21	0	1	10
Time of virological failure – before study (months)	67,35	31	1	275	5	31	129
Time to undetectable VL (months) - after recruitment	5.8(±4.2)	4	1	15	2	4	10
Time of follow up (months)	14.7 (±11.7)	12	6	42	6,5	12	16,5
CD4 in follow up	194 (±134)	140	6	485	98	140	299

Supplementary Information 1

Statistical analysis

Time of diagnosis	N	%
0-12months	7	41,2
13 a 60 months (5 years)	4	23,5
61 a 120 months (5-10 years)	1	5,9
> 121 months (> 10 years)	5	29,4
Total	17	100,0

Time of virological failure before recruitment

N	Valid	17
	Missing	0
Mean		67,3529
Median		31,0000
Percentiles	25	5,0000
	50	31,0000
	75	129,5000

Time to undetectable VL (total)	N	%
0-12 months	6	35,3
13 a 60 months (5 years)	6	35,3
61 a 120 (5-10 years)	3	17,6
> 121 months (> 10 years)	2	11,8
Total	17	100,0

Time to undetectable VL (years)

N	Valid	15
	death	2
Media		5,3333
Median		1,0000
SD		6,76827
Minimum		,00
Maximum		21,00
Percentis	25	,0000
	50	1,0000
	75	10,0000

Supplementary Information 1

Statistical analysis

Time to undetectable VL (after the recruitment)	N	%
0-2 months	4	35,3
2-4 months	4	35,3
4-10 months	5	17,6
10-15 months	2	11,8
Total	17	100,0

Time to undetectable VL (after the recruitment) - months

N	Válido	15
	Ausente	2
Media		5,8000
Median		4,0000
SD		4,24601
Minimum		1,00
Maximum		15,00
Percentis	25	2,0000
	50	4,0000
	75	10,0000

Outcomes for CrAg-positive patients after the recruitment

- The time of virological failure had a media of 31 months, with IQI 5-129.
- 15/17 (88.2%) were retention in care, 12/17 (70.5%) at follow up in the original service; 3/17 (17.6%) at follow up other facilities
- 2/17 (11.7%) died
- 10 (59%) subjects had the ART changed because of virological failure
- 6/17 (35.3%) had viral resistance, confirmed by genotyping
- 6/17 (35.3%) weren't in regular use of ART during the last year of follow up and 1/15 wasn't adherent for the last three months
- Preemptive treatment: 9 with fluconazole; 4 with itraconazole and 1treated with amphotericin + fluconazole
- 11/17 (64.7%) used the recommended treatment for at least 10 weeks

As outcomes:

- 2/17 (11.7%) deaths;
- 13/17 (76,4%) had opportunistic diseases during follow up
  - Neurotoxoplasmosis: 6/17 (35.3%)
  - Histoplamosis: 4/17 (23.5%)
  - Tuberculosis: 3/17 (17.6%)
  - PCP: 2/17 (11.8%)
  - Moniliasis: 4/17 (23.5%)
  - CMV (2/17 (11.8%)
  - Herpes Zoster: 3/17 (17,6%)

Supplementary Information 1

Statistical analysis

Characteristics of CrAg-positive individuals and follow-up.

Patient	Duration of detectable viral load T1[1] (months)	Follow-up time (months)	Cryptococcal Meningitis	T1 CD4 (cells/µl)[1]	T1 viral load (copies/ml)[1]	Regular ART on T1[1]	T2 CD4 (cells/µl)[2]	In care	T2 viral load (copies/ml)[2]	Time to viral suppression after study (months)	Regular ART on T2[2]	Treatment - definitive - or pre-emptive
1	121	42	No	28	621,269	Yes	485	Yes	<40	7	Yes	Fluconazole
2	4	42	Yes	199	135	Yes	425	Yes	183	12	Yes	Amphotericin+Fluconazole
3	5	28	No	130	10,383	Yes	332	Yes	<40	15	Yes	Fluconazole
4	10	17	No	126	9,270	Yes	47	Yes	<40	10	Yes	Itraconazole
5	5	16	No	7	140,766	Yes	184	Yes	<40	2	Yes	Fluconazole
6	93	15	No	75	378,294	No	140	Yes	<40	4	Yes	Itraconazole, irregular[3]
7	257	14	No	36	4,218	No	132	Death	<40	3	Yes	Fluconazole, irregular
8	148	12	No	10	54,376	No	125	Yes	125,559	10	No	Fluconazole
9	31	12	No	37	52,2170	Yes	124	Yes	<40	6	Yes	Fluconazole
10	59	7	No	27	25,826	Yes	243	Yes	<40	2	Yes	No use
11	59	8	No	31	2,423,874	No	6	Death	1,112,547	-	No	No use
12	192	7	No	56	50	No	72	Yes	<40	4	Yes	Itraconazole
13	2	7	No	20	337,560	No	117	Yes	<40	4	Yes	Fluconazole
14	1	6	No	96	112,513	Yes	290	Yes	<40	6	Yes	Itraconazole
15	138	6	No	97	121	Yes	97	Yes	121	-	Yes	No use
16	13	6	No	158	<40	Yes	202	Yes	<40	1	Yes	No use
17	7	6	No	143	228	Yes	308	Yes	<40	1	Yes	Fluconazole

[1]At time of screening (first evaluation).

[2]At time of re-evaluation (second evaluation).

[3]Irregular use of pre-emptive treatment: <10 weeks.

Supplementary Information 1

Statistical analysis

Characteristics of ART and virological control in CrAg-positive individuals

Patient	Time of HIV diagnosis at T1[1] (months)	Duration of detectable viral load T1[1] (months)	Time to ART initiation after diagnosis (months)	Adherent to ART during the last year before T1[1]	Time of ART Resumption at T1[1] (days)	Time to viral suppression after study (months)	ART regime at T1[1]	Regular ART during last year of follow-up	Viral Resistance	ART at T2[2]
1	121	121	118	No	150	7	TDF + 3TC + EFZ	Yes	No	TDF + 3TC + ATV + RTV
2	4	4	1	Yes	118	12	TDF + ATV + RTV	No	No	TDF + 3TC + DRV + RTV
3	5	5	3	Yes	48	15	TDF + 3TC + EFZ	Yes	yes	TDF + 3TC + DRV/R + DTG
4	10	10	4	Yes	175	10	TDF + 3TC + EFZ	Yes	yes	TDF + 3TC + DRV/R + DTG
5	5	5	2	Yes	96	2	TDF + 3TC + DTG	Yes	No	TDF + 3TC + DTG
6	93	93	21	No	50	4	TPV + TDF + 3TC + RTV + DTG	No	yes	TDF + 3TC + DRV/R + DTG
7	257	257	175	No	125	3	TDF + 3TC + RTV + DTG + DRV	death	No	TDF + 3TC + DRV/R + DTG
8	148	148	64	No	194	10	TDF + 3TC + ATV + RTV + DTG	No	yes	TDF + 3TC + ATV + RTV + DTG
9	31	31	1	No	54	6	TDF + 3TC + EFZ	Yes	No	TDF + TC + EFZ

10	59	59	0	No	365	2	TDF + 3TC + EFZ	No	yes	TDF + 3TC + DRV/R + DTG
11	59	59	0	No	324	-	TDF + 3TC+ ATV + RTV	death	No	TDF + 3TC + ATV + RTV
12	192	192	103	No	0	4	TDF + 3TC + RTV + DRV + DTG	No	yes	TDF + 3TC + DTG
13	2	2	1	No	0	4	não utilizava tarv	Yes	No	TDF + 3TC + DRV + RTV
14	1	1	0	Yes	35	6	TDF + 3TC + DTG	Yes	No	TDF + 3TC + DTG
15	138	138	67	No	126	-	TDF + 3TC + ATV + RTV	No	No	TDF + 3TC + ATV + RTV
16	18	13	2,7	Yes	475	1	TDF + 3TC + DTG	Yes	No	TDF + 3TC + DTG
17	7	7	0	Yes	209	1	TDF + 3TC + EFZ	Yes	No	TDF + 3TC + DTG

[1]At time of screening (first evaluation).

[2]At time of re-evaluation (second evaluation).

Supplementary Information 1

Statistical analysis
